# Alterations in neuromuscular function in girls with generalized joint hypermobility

**DOI:** 10.1186/s12891-016-1267-5

**Published:** 2016-10-03

**Authors:** Bente Rona Jensen, Jesper Sandfeld, Pia Sandfeld Melcher, Katrine Lyders Johansen, Peter Hendriksen, Birgit Juul-Kristensen

**Affiliations:** 1 Department of Nutrition, Exercise and Sport, Integrative Physiology, Biomechanics and Motor Control Laboratory, University of Copenhagen, Copenhagen, Denmark; 2 Research Unit for Musculoskeletal Function and Physiotherapy, Institute of Sports Science and Clinical Biomechanics, University of Southern Denmark, Odense, Denmark; 3 Department of Health Sciences, Institute of Occupational Therapy, Physiotherapy and Radiography, Bergen University College, Bergen, Norway; 4 Department of Neurology, Odense University Hospital, University of Southern Denmark, Odense, Denmark

**Keywords:** Hypermobile, Knee, EMG, Muscle activation, Rate of force development

## Abstract

**Background:**

Generalized Joint Hypermobility (GJH) is associated with increased risk of musculoskeletal joint pain. We investigated neuromuscular performance and muscle activation strategy.

**Methods:**

Girls with GJH and non-GJH (NGJH) performed isometric knee flexions (90°,110°,130°), and extensions (90°) at 20 % Maximum Voluntary Contraction, and explosive isometric knee flexions while sitting. EMG was recorded from knee flexor and extensor muscles.

**Results:**

Early rate of torque development was 53 % faster for GJH. Reduced hamstring muscle activation in girls with GJH was found while knee extensor and calf muscle activation did not differ between groups. Flexion-extension and medial-lateral co-activation ratio during flexions were higher for girls with GJH than NGJH girls.

**Conclusions:**

Girls with GJH had higher capacity to rapidly generate force than NGJH girls which may reflect motor adaptation to compensate for hypermobility. Higher medial muscle activation indicated higher levels of medial knee joint compression in girls with GJH. Increased flexion-extension co-activation ratios in GJH were explained by decreased agonist drive to the hamstrings.

## Background

Generalized joint hypermobility (GJH) is a condition where the individual’s joint has an exaggerated ability to exceed beyond the normal range of motion, due to increased connective tissue flexibility and/or capsular or ligamentous looseness [1, 2]. The prevalence of GJH is both age and sex-related but varies significantly in the published reports (varying from 8 to 30 % for children [3, 4]), depending on testing procedures and the cut-off point used to define the presence of GJH. Adolescents and adults within specific groups, such as ballet dancing, gymnastics and musicians have shown high prevalence (27–90 %), thus indicating an advantage of being hypermobile for performance (e.g. [5, 6]). However, GJH seems also to be a disadvantage due to an increased risk of knee joint injuries [7–9], especially in contact activities [10]. Knee hyperextension and side-to-side differences in tibiofemoral translation are predictive of future ACL- injury status [11]. Furthermore, hypermobility is anticipated to be associated with increased risk of musculoskeletal disorders, especially osteoarthritis later in life (e.g. [12]). Children with GJH exhibit a high prevalence of arthralgia with the knee being the most frequently reported joint with pain, and a large proportion report exercise related joint pain [13]. In addition, GJH is a predictor for development of future joint pain, symptoms and injuries [8–10].

Explosive muscular force has been defined as the capability to increase contractile force (or torque) from resting level or near resting level as quickly as possible, and it can be quantified as rate of force (or torque) development, i.e. increase per time [14]. The ability to rapidly produce muscular force is important for performance in sport and in daily activities where the time frame available for the development of force is limited. These activities include, for example, running and jumping as well as, balance challenging tasks, locomotion and response to mechanical perturbation [15–17]. Rate of force (or torque) development is determined by neural and contractile determinants; this includes neural drive to agonist and antagonist muscles, muscle strength, muscle-tendon unit stiffness and morphological factors such as fiber type composition [14, 18]. Our knowledge regarding rate of force development in individuals with GJH is limited to one study on non-symptomatic female adults with GJH that showed faster knee extension rate of force development in female adults with GJH compared to a reference group [19]. The ability to rapidly produce muscular force in children with GJH and non-hypermobile girls (NGJH) has not been studied.

Joint loads during locomotion are highly dependent on internal forces, i.e. muscle activation and forces from non-contractile tissue, and to a lesser extent on external forces. Little is known about muscle activation patterns in GJH. Reduced agonist activation during isometric knee flexion in 90° and a larger co-activation ratio due to reduced agonist activation during isometric knee flexion in 90° were recently reported in young children and adults with GJH (full knee extension corresponding to 180°) [20]. However, it is not known if this change in knee muscle activation pattern can be generalized to more extended knee positions. To explore this, in the present study we investigated knee muscle activation and co-activation in less flexed knee angles, in addition to measurement of rate of torque development (RTD).

The purpose of this study was to expand knowledge on neuromuscular performance and muscle activation strategy of hypermobile girls during submaximal isometric, explosive and maximum voluntary contractions of the knee muscles.

## Methods

### Subjects

Girls with GJH and NGJH girls, aged 14–15 years, were recruited randomly from a cohort of schoolchildren (for details, see [21]). Hypermobility was assessed according to the Beighton score (range 0–9) [22]. Inclusion criteria for girls with GJH were Beighton score ≥ 6, at least one hypermobile knee, and no or minimum knee pain within the latest week. Exclusion criteria for both groups were previous or current knee trauma or knee pain. Sixteen girls with GJH (Beighton score 6.8 ± 0.7, no or minimum knee pain) and 11 healthy NGJH girls (Beighton score 1.7 ± 1.8) participated. Their ages, body masses and heights were 14.0 ± 0.0 years, 57.7 ± 9.1 kg, 1.66 ± 0.05 m (GJH) and 14.3 ± 0.5 years, 57.2 ± 4.5 kg, 1.66 ± 0.04 m (NGJH), respectively. Both groups were physically active. They performed physical exercise 3.7 h per week (GJH) and 4.1 h per week (NGJH). Both groups filled out the Knee Injury and Osteoarthritis Outcome score for Children (KOOS-Child) [23]. No between groups differences in KOOS-Child were found (for details see [21]).

### Procedures

The experimental setup was the same as in our recent study [20]. Briefly, the subjects performed isometric knee flexion and extension contractions in a sitting position with the thigh horizontal. The subjects were secured to the chair with Velcro straps. Contractions were performed with their dominant leg.

Three types of contractions were performed: Maximum isometric knee flexions and extensions, sustained isometric submaximal contractions at 20 % MVC and explosive isometric contractions. A familiarization procedure was performed before each type of contraction. Maximum knee flexions were performed with knee angles of 90°, 110° and 130° and knee extensions were performed with a knee angle of 90°. Three repetitions were performed in each position and these knee contractions were separated by 1–2 min rest. If the last contraction was the highest in a certain position, 1 or 2 additional contractions were performed. The order of knee angle was randomized between subjects. The contraction with the highest exerted force in each position was selected as maximum voluntary contractions (MVCs) and used for calculation of target levels for the submaximal contractions. The submaximal isometric knee flexions (knee angles 90°, 110° and 130°) and knee extensions (knee angle 90°) were performed at 20 % MVC for 30 s each. Three submaximal contractions were performed in each direction and for each knee angle. Visual force feed-back (Pico-scope 2205; Pico Technology, UK) was provided to the subjects throughout the contractions. In addition, the subjects were guided verbally during the contractions. One-minute rest was allowed between contractions. Average values of the three contractions for each trial are reported. Finally, the subjects performed explosive isometric knee flexions at a knee angle of 90° while sitting. The task was to increase the force as fast as possible, upon a randomly delayed (0–10 s) visual cue, to a level above 50 % MVC. Visual force feedback was given to the subjects during the task. The explosive knee flexions were repeated 5 times.

### Measurements

Force was measured with a strain-gauge force transducer (PMH Electronic, Denmark) connected to a strap surrounding the ankle just proximal to the lateral malleolus. The orientation of the force measurements was perpendicular to the axis of the lower leg. The moment arm was measured as the distance from the middle of the ankle strap to the center of rotation of the knee. Surface electromyography (EMG) was measured from two knee extensor muscles (mm. vastus lateralis (VL) and medialis (VM)) and four knee flexor muscles (m. biceps femoris (BF), m. semitendinosus (ST) and mm. gastrocnemius lateralis (GL) and medialis (GM), using surface electrodes (Ag/AgCl electrodes, 720-01-K; Medicotest, Denmark). The electrodes were attached to the skin in a bipolar configuration with an inter-electrode center distance of 2 cm, and in line with the muscle fiber orientation. The skin was carefully shaved and rubbed with sandpaper and alcohol before attachment of the electrodes to ensure an inter-electrode resistance < 10 KOhm. The EMG signals were pre-amplified (x25), high pass filtered at 10 Hz and low pass filtered at 400 Hz and amplified (Logger Technology, Sweden).

EMG and force were sampled (Scope, Version 2.3, Data Translation) at a sampling rate of 1 kHz (DT BNC Box, USB Series, Data Translation, USA).

### Data analysis

Muscle strength was determined as the highest 1-s values measured during the maximum knee flexions and knee extensions. The 1-s values were calculated based on 10 successive 100-ms root-mean-square values. Muscle strength (torque) was normalized to body mass. For the submaximal contractions, EMG amplitudes were calculated as the root-mean-square (RMS) values (100 ms segments). EMG_RMS_ measured during the submaximal contractions was normalized to maximum EMG values measured during MVC contractions and expressed as %EMGmax. Average EMG_RMS_ was calculated for each 30-s contraction, excluding the first and the last 5 s from the analysis. Extensor muscle activation was calculated as average activation of VM and VL, flexor muscle activation as the average activation of BF and ST, and as average activation of GM and GL. Extension-flexion co-activation ratio was calculated as average antagonist EMG_RMS_ x average agonist EMG_RMS_^−1^ × 100 (%). Furthermore, medial-lateral co-activation ratio was calculated as average medial EMG_RMS_ × average lateral EMG_RMS_^−1^ × 100 (%). For the explosive knee flexions, early RTD was calculated as slope of the torque curve from 15 to 35 % of maximum torque as measured during MVC. The fastest trial is reported and expressed as Nm/s. Data were analyzed with a MATLAB algorithm.

### Statistics

Statistical analyses were performed in Minitab (MTB version 16, Minitab, USA). A general linear model (GLM), with condition (GJH or NGJH) and knee angle as fixed factors was used to identify differences. Unpaired T-test was used to identify group differences in knee extension strength. Data are presented as group mean +/− SD or grand average across angles in the text, and as means +/− SE in figures. Statistical difference was set at *P* < 0.05.

## Results

**Fig. 1:**
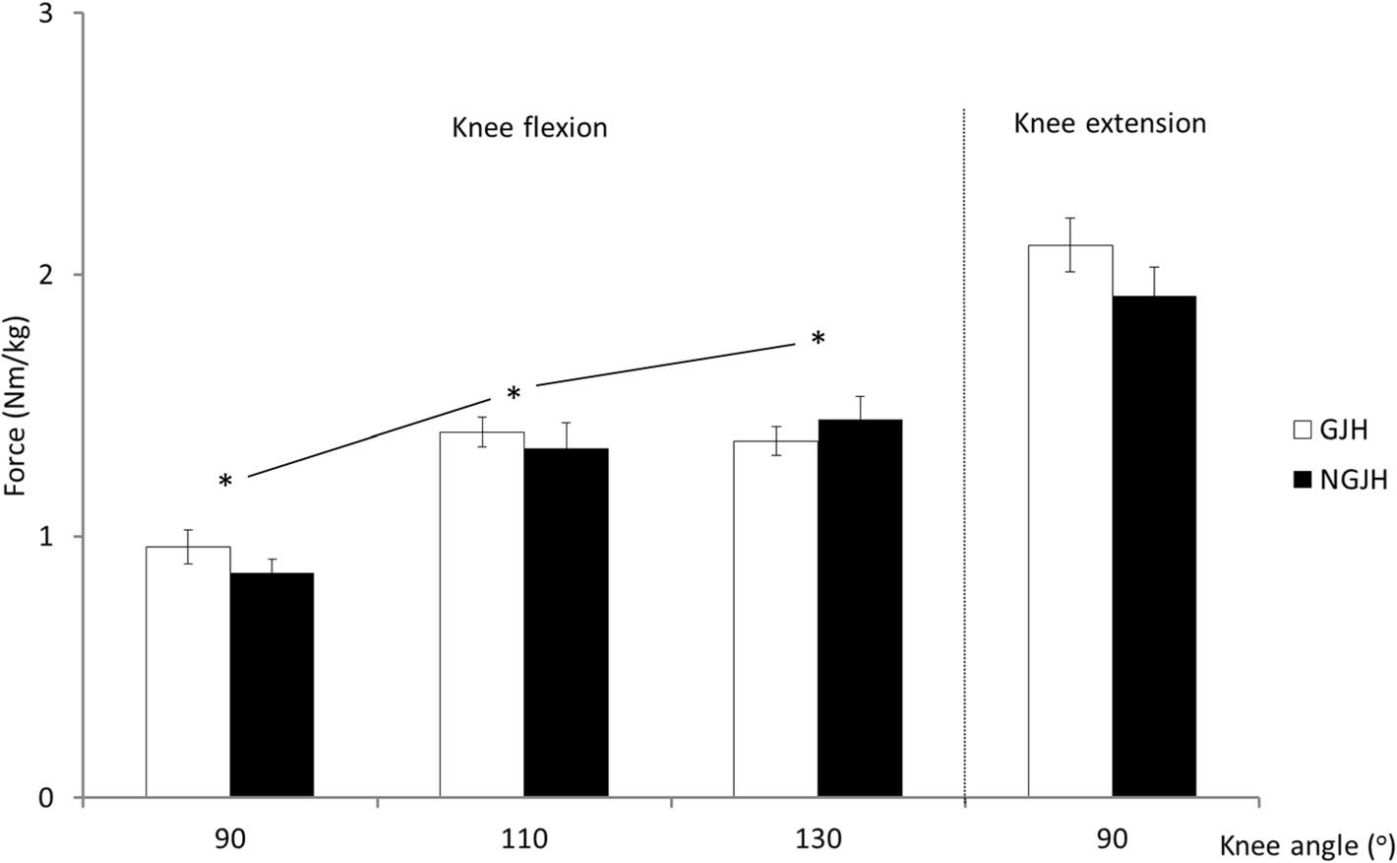
Muscle strength during isometric knee flexion and knee extension. * indicate differences between knee flexion angles (main effect across the three knee angles). Mean (SE)

Knee joint MVC in girls with GJH did not differ from NGJH girls at any knee flexion angle (*P* = 0.481) or during knee extension (*P* = 0.210). Knee flexion MVC increased with increasing knee angle across both groups (main effect) (*P* < 0.001) (Fig. 1).

**Fig. 2:**
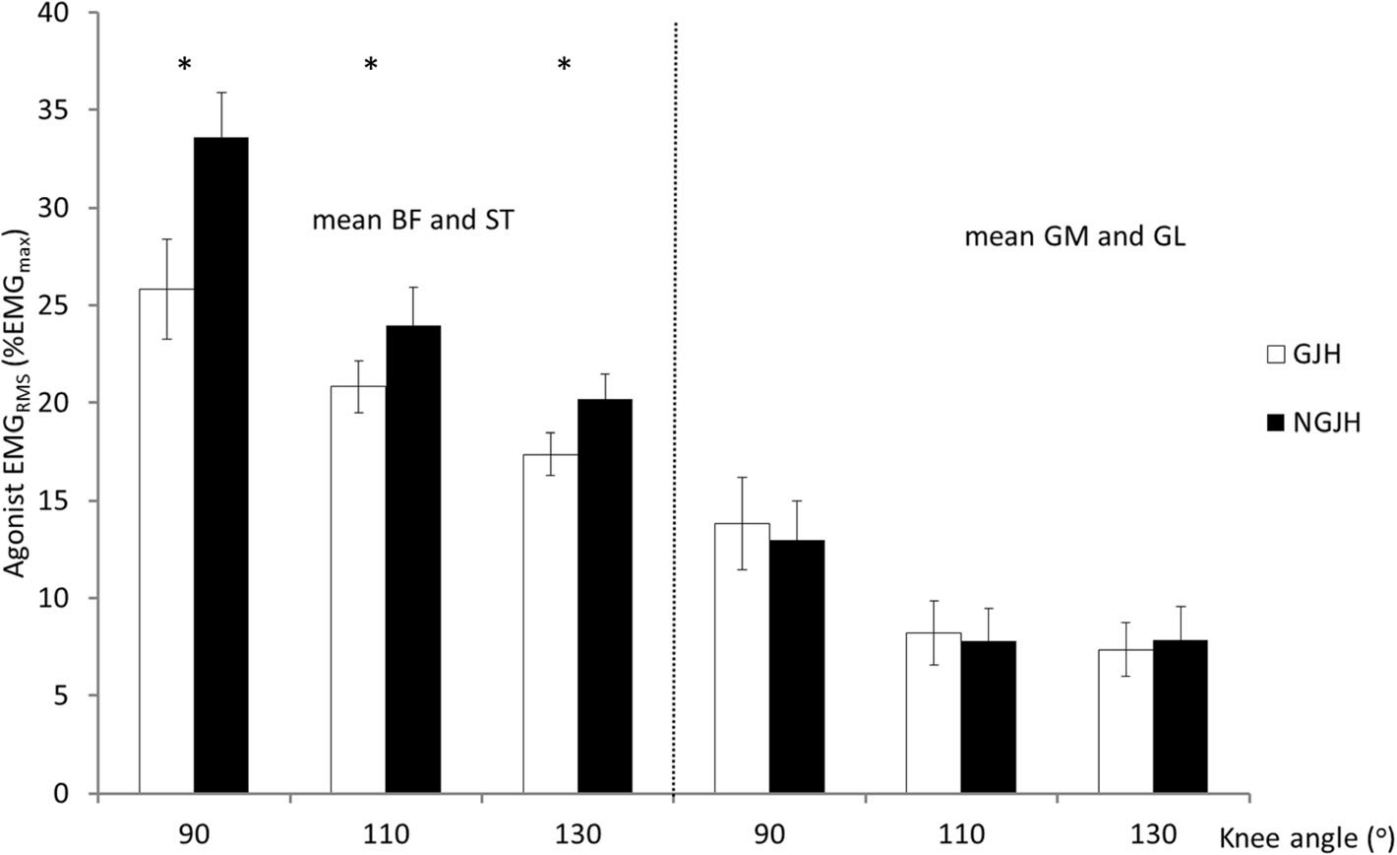
Agonist EMG during knee flexion at 20%MVC. BF: m. biceps femoris. ST: m. semitendinosus. GM: m. gastrocnemius medialis. GL: m. gastrocnemius lateralis. * indicate differences between groups. Mean (SE)

During knee flexion at 20 % MVC, hamstring muscle activity was significantly lower (*P* < 0.001) in girls with GJH (grand average 21.3 % EMGmax) relative to NGJH girls (grand average 25.9 % EMGmax). No between groups difference was found for m. gastrocnemius (*P* = 0.793) (Fig. 2). Main effects of knee angle, with decreasing activation of the hamstring muscles and the gastrocnemius muscles with increasing knee flexion angles were found (hamstring: *P* < 0.001, mm. gastrocnemius: *P* < 0.001)). Antagonist muscle activation during knee flexions did not differ between girls with GJH (grand average: 3.0 % EMGmax) and NGJH girls (grand average: 3.1 % EMGmax) (*P* = 0.730). During knee extension at 20 % MVC, no between groups differences were found.

**Fig. 3:**
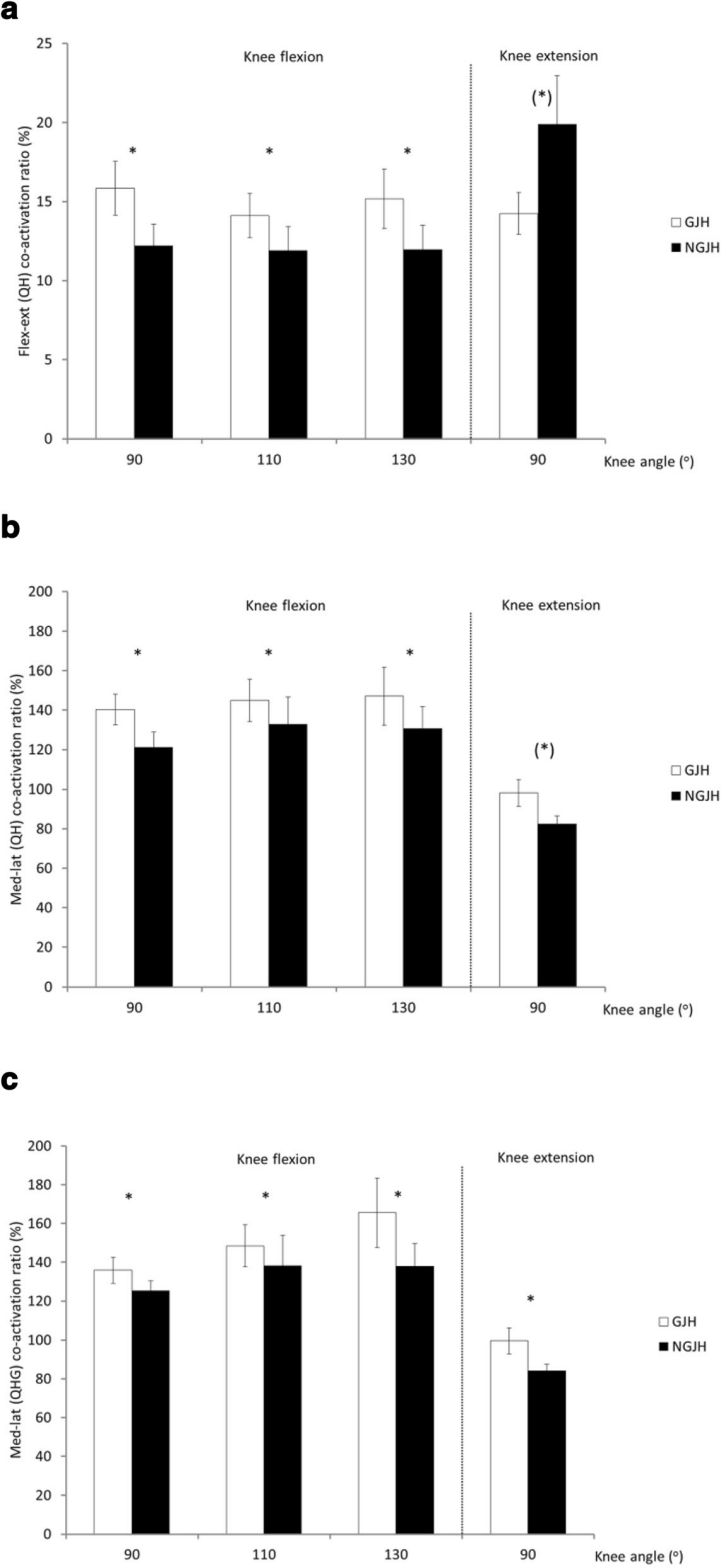
Co-activation ratio during knee flexion and extension. **a** Flexion-extension. **b** Medial-lateral including two muscle groups (quadriceps and hamstrings, QH). **c** Medial-lateral including three muscle groups (quadriceps, hamstrings and gastrocnemius, QHG). * indicate differences between groups. (*) indicates *P* < 0.1. Mean (SE)

Flexion-extension co-activation ratios, measured during knee flexions and calculated as antagonist activation (VL, VM) divided by agonist activation (BF, ST), were higher in girls with GJH (grand average: 15.0 %) than in NGJH girls (grand average: 12.0 %) (*P* < 0.001) (Fig. 3a). No main effect of knee angle was found (*P* = 0.179). In contrast, flexion-extension co-activation ratios during 90° knee extension tended to be higher in girls with NGJH than in GJH.

Medial-lateral co-activation ratios, measured during knee flexion and calculated as medial muscle activation divided by lateral muscle activation including quadriceps and hamstring muscles, were higher for GJH (grand average: 144.1 %) than for NGJH (grand average: 128.3 %), (*P* = 0.015) (Fig. 3b). Also, medial-lateral co-activation ratios, including quadriceps, hamstrings and gastrocnemius muscles, were higher for GJH (grand average: 149.9 %) than for NGJH (grand average: 133.8 %) (*P* = 0.019) (Fig. 3c). Higher values of medial-lateral co-activation ratio in GJH than in NGJH were consistent across contraction type.

**Fig. 4:**
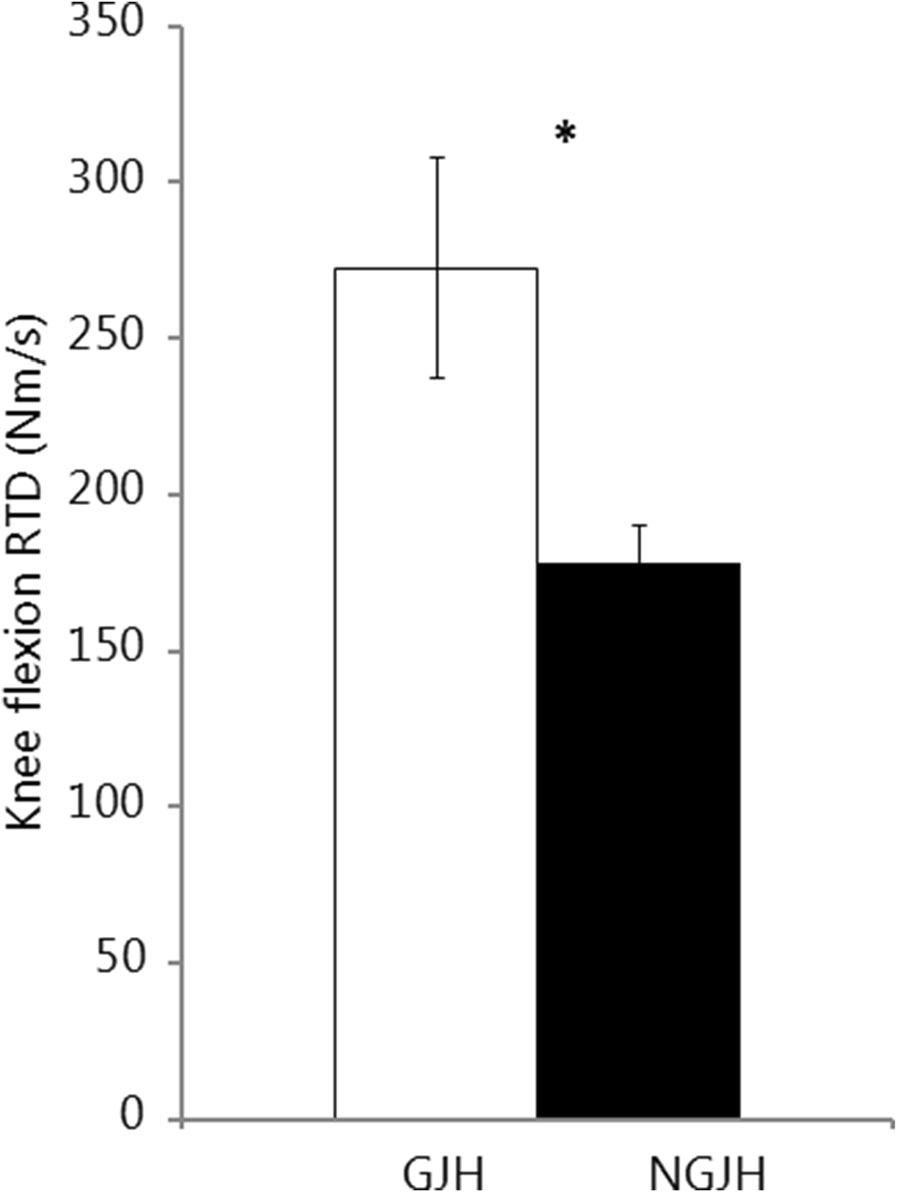
Early rate of torque development (RTD). Mean (SE). * indicate differences between groups. Mean (SE)

Girls with GJH had higher RTD in knee flexion than NGJH girls (Fig. 4). Thus, girls with GJH had on average 53 % faster RTD than NGJH girls (*P* = 0.037).

## Discussion

A main finding of the present study was that the capacity for early rapid torque generation during knee flexion was higher in girls with GJH than NGJH girls, despite no group differences in muscle strength. Furthermore, girls with GJH had higher medial-lateral co-contraction ratios during submaximal isometric contractions of the knee muscles, and had reduced activation of the hamstring muscles during submaximal knee flexion as compared to NGJH girls.

Girls with GJH had higher neuromuscular capacity to rapidly generate torque, i.e. greater absolute RTD, during isometric knee flexion than NGJH girls, despite no between group differences in isometric knee flexion strength. Explosive force production should be seen as a sequential process across time since the relative contribution of the neural and contractile predictors change throughout the contraction [14]. In the present study RTD was measured in the early phase of the contraction where neural and muscle intrinsic factors are of particular importance, whereas the importance of MVC is less pronounced [14]. It is therefore likely that neural and/or muscle intrinsic factors can explain the greater early absolute RTD in the GJH group.

An alternative theory to explain the higher RTD is that girls with GJH use a more optimal joint position for rapid force generation in the isometric knee flexion test compared to NGJH girls, or in other words girls with GJH operate closer to the optimal region of the length-tension curve than NGJH girls. An association between relative muscle length and rate of force development in children with cerebral palsy was suggested recently as a possible underlying mechanism for understanding why these children have a reduced range of motion and reduced rate of force development compared to typically developing children [24].

In accordance with the present results, Mebes at al. found higher knee extensor rate of force development in adult women with GJH than in individuals with normal range of movement. Furthermore, rate of force (or torque) development is important for jumping height [25] and 10 year old children with GJH perform better in vertical counter movement jump than NGJH children [26]. Taken together, these results support the idea that GJH facilitate high levels of RTD in general. Adaptation of early force generating capacity during physical training has been documented [27]. The majority of the participants in the present study were active in sports, but no differences between groups were found [21]. Thus, between group differences in physical training status cannot explain the present increased early RTD in girls with GJH.

RTD is considered to be important for joint stabilization [16], and individuals with GJH require more neuromuscular joint stability due to their joint laxity to protect their joints. This suggests that a higher level of RTD in GJH is a motor adaptation strategy to compensate for their joint hypermobility (laxity), rather than a consequence of GJH. The specific explanation for why girls with GJH had higher early absolute RTD than NGJH girls requires further investigations, and this issue is outside the scope of the present study.

Despite the increased ability to stabilize the knee, GJH individuals are anticipated to have a higher risk of joint osteoarthritis, and have been found to have increased risks of injury and pain [9, 10]. In the present study medial knee muscle activation was higher than lateral knee muscle activation in both groups. However, greater values of medial-lateral co-activation ratios, towards higher medial muscle activation were found for girls with GJH compared to NGJH girls, indicates higher levels of medial joint compression in girls with GJH, due to a markedly asymmetric loading of the knee in this group. This specific muscle activation pattern in GJH girls was also measured during two-legged and one-legged balance tasks in our recent study where the same group of girls with GJH participated [21]. Both tasks are characterized as static or semi-static tasks.

Compressive knee load is assumed to play a significant role in development of knee osteoarthritis [28, 29]. Furthermore, a recent longitudinal study provided evidence that muscle activation strategy during gait is related to progression of medial site knee osteoarthritis which is the most common site of knee osteoarthritis. Thus, a greater duration of medial muscle co-contraction and medial relative to lateral co-contraction correlated positively with loss of medial cartilage volume, whereas lateral muscle co-contraction correlated inversely with cartilage loss [29]. Based on these findings it seems likely that GJH specific muscle activation strategy, with higher medial muscle activation, plays a role in the increased risk of joint symptoms in persons with GJH.

Our findings of decreased activation of the hamstring muscles during 90° knee flexion contraction confirmed our previous results in 10 year old children and in adults [20]. The present study adds new knowledge regarding the significance of knee angle for activation of the hamstring muscles.

One limitation of this study is that muscle activation strategy was only determined during isometric contractions. A second limitation is the small sample size. However, significant group differences in muscle activation strategy, even with this small group size, were found. Finally, muscle morphological factors that might influence force generation were not included in the study.

## Conclusion

Higher early RTD during knee flexion contraction in girls with GJH indicating improved ability to stabilize the knee was found. We have demonstrated alterations in knee muscle activation strategy in girls with GJH. Firstly, higher levels of medial muscle activation during isometric contractions of the knee muscles were found in girls with GJH which may be associated with increased risk of joint symptoms later in life. Secondly, our study confirmed that knee muscle activation strategy during knee flexions in girls with GJH is changed relative to NGJH girls towards less activation of the hamstring muscles in flexed knee positions. Thus, the increased flexion-extension co-activation ratios in girls with GJH could be explained by decreased agonist drive to the hamstrings. The difference in hamstring muscle activation strategy was less pronounced in more extended knee positions.

## Abbreviations

ACL: Anterior cruciate ligament
BF: m. biceps femoris
EMG: Electromyography
GJH: Generalized joint hypermobility
GL: m. gastrocnemius lateralis
GM: m. gastrocnemius medialis
MVC: Maximum voluntary contraction
NGJH: No generalized joint hypermobility
QH: Quadriceps and hamstrings
QHG: Quadriceps hamstrings and gastrocnemius
RFD: Rate of torque development
RMS: Root mean square
ST: m. semitendinosus
VL: m. vastus lateralis
VM: m. vastus medialis
